# General practice nurse trainees’ perspectives on general practice nursing as a career choice: qualitative findings from a vocational training scheme in the United Kingdom (UK)

**DOI:** 10.1186/s12875-023-02165-8

**Published:** 2023-10-21

**Authors:** Robin Lewis

**Affiliations:** College of Health, Wellbeing and Lifesciences; Sheffield Hallam University, Sheffield , S10 2BP UK

**Keywords:** General practice, Primary care workforce, Education and training, General practice nursing, Career pathways, Continuing professional development

## Abstract

**Background:**

There is a shortage of general practice nurses worldwide to deal with an ever-increasing workload, and the need to attract new staff into general practice nursing is therefore vital. As part of this, a one-year Vocational Training Scheme (VTS) for new to general practice nurses was developed in 2020 by the South Yorkshire Primary Care Workforce and Training Hub.

**Methods:**

The aim of the study was to examine the VTS trainees’ views on general practice nursing as a career. A pragmatic, convenience sample of trainees was recruited. Of the 21 trainees, 17 agreed to take part in the study. Data were collected from the trainees using a series of four regular, timed, online focus groups designed to follow the trainees’ trajectory on the programme over a 12-month period. The data were analysed using framework analysis.

**Results:**

The timed nature of the focus groups meant that the analysis of the data was linked to the trainees’ trajectory over the course of the year. Three themes were generated from the data: ‘*pathways into general practice’*; ‘*learning to be a GPN’;* and *‘the future GPN’.* In theme one, the trainees talked of the difficulties in accessing general practice as a new graduate, specifically the need for prior experience and how to get it. In the second, the transition to being a general practice nurse was discussed, and the expectation of being able to ‘hit the ground running’ once in post. The new graduate participants were also concerned over the opportunities for clinical supervision and support in the role after the programme. Finally, the participant s expressed concern over future opportunities for professional development and the prospects for a long-term career in general practice.

**Conclusion:**

To address the worldwide workforce ‘crisis’ in general practice nursing, sustainable career pathways are needed to encourage new graduate nurses to consider working in general practice. Starting at university, changing the culture and providing the necessary infrastructure to support ongoing professional development in general practice nursing are key to its success.

## Background

Train people well enough so they can leave… treat them well enough so they don’t want to.

Attributed to Sir Richard Branson.

In recent years there has been a worldwide shift in healthcare policy from a hospital-based secondary care focus, towards a greater emphasis upon primary care and public health. In the United Kingdom (UK), two government documents, the ‘Five Year Forward View’ [1] and ‘Long-Term Plan’ [2] outlined a concerted shift from a secondary to primary care focus in the management of older adults with LTCs. Mirrored worldwide, it is estimated that in the UK 58% of people over the age of 60 are living with at least one long-term condition (LTC), with the majority of these individuals being managed in primary care by General Practice Nurses (GPNs) [3]. As people live longer, but not necessarily better, there is an ever-increasing demand for primary care services worldwide [4]. Post-COVID 19, the provision of primary care worldwide is continually under the spotlight [5].

There is however a worldwide recruitment and retention crisis in general practice nursing (GPN) [6–10]. The Queen’s Nursing Institute (QNI) in the UK identified that approximately 33% of GPNs were likely to have retired by 2021 [11]. Exacerbated by the COVID-19 pandemic, a significant proportion of this critical mass of experienced GPNs have now disappeared from the general practice workforce [12].

The increased emphasis upon the management of LTCs in primary care means that since there is no clear recruitment and retention strategy in place to increase the numbers of GPNs ‘at scale’, post-COVID there is a ‘perfect workforce storm’ brewing. This will consist of an acute shortage of GPNs at the very time when the workload in primary care is increasing exponentially [11, 13, 14].

## Literature review

In recent years, there have been various attempts to address the GPN workforce crisis, however attracting nurses into primary care has always been a challenge. General practice has not been considered a suitable ‘first post’ destination for new graduate nurses [15–18]. It has been argued [15] that at least part of this antipathy has been caused by the continued focus upon secondary and acute care in the undergraduate (UG) nursing curriculum. The absence of much primary care content, together with a shortage of general practice placements for student nurses has meant that many student nurses still do not know what general practice nursing is, or what it has to offer [19]. It may be argued that Higher Education Institutions (HEIs) worldwide are still slow to reflect the increased emphasis upon primary care in terms of developing both clinical placements and curriculum content [20].

Unlike medicine, there has been no culture of student nurses spending time on placement in general practice, and consequently there has been a perceived lack of understanding amongst GPs regarding the nature of the undergraduate nursing curriculum and what it has to offer [16]. There have been a number of initiatives over the years designed to address these issues and increase student nurse access to general practice placements [21, 22]. In a number of areas within the United Kingdom (UK), GPs were commissioned to provide placements for student nurses. These schemes, funded by Health Education England (HEE) were known as Community Education Provider Networks (CPENs) [23] or Advanced Training Practices (ATPs) [24].

General practices in the UK are usually owned by either a single General Practitioner (GP) or a group of GPs, and as with other countries such as Australia and New Zealand they operate within an independent ‘small business’ model or as part of a larger corporate chain. Income is primarily generated through a combination of payment models with variable ‘fees-for-service’ arrangements [22, 24]. As Bauer & Bodenheimer [22] note, health care reformers have highlighted the need to move away from ‘fees for service’ towards a system of payment which rewards quality of care rather than simply the volume of activity. The reform of payment for primary care service, whether through private medical insurance, publicly funded insurance, or direct taxation is vital to the future of general practice. Indeed, the way in which primary care is funded plays a significant role in the recruitment and retention issues identified in general practice nursing staff.

In the UK, GPs are subcontracted to provide a range of services to the National Health Service (NHS). GPNs in the UK are employed by the ‘business’ and not the NHS, and as a result, GPs have been reluctant to invest in education and training for new GPNs, much preferring to recruit experienced nurses who can ‘hit the ground running’ [16, 24]. In the UK, GPN recruitment has been predicated upon access to a (rapidly diminishing) pool of older, experienced GPNs who may be simply ‘poached’ from other practices as required [24]. In the long term, this had the effect of dissuading younger, new graduates from applying for GPN posts.

It became clear that whilst they have made a difference, the CPEN/ATP schemes were not going to be able to deliver the numbers of new GPNs that were required to address the predicted shortfall [25]. Evidence has suggested that preceptorship type programmes were required to support the transition of new to general practice nurses (NTGPNs) into the role, and in doing so, address GPs reservations [25–31].

If NTGPNs are to be successfully recruited and retained therefore, it is argued that there needs to be a significant cultural shift in the way that GPN education and development is organised [32, 33]. Since they are not employed by the NHS, attempts at addressing the need for GPN continuing professional development in the UK have often been thwarted by the reluctance of GP employers to fund education and training programmes [34].

Unlike their medical counterparts, there is still no formal, nationally accredited entry qualification and associated training programme for GPNs in the UK [32]. This situation is mirrored worldwide in other developed countries such as Australia, USA, and New Zealand [33, 34]. In an attempt to address this situation, a GPN Fellowship scheme was developed in the UK. Arising out of the Long Term Plan [2], the GPN Fellowship committed to provide a two-year programme of training for NTGPNs. This has begun to address some of the systemic problems within the provision of education for GPNs, and to act as an incentive for new to general practice nurses to actively consider a career in general practice.

### The VTS programme

The SY VTS programme is a one-year vocational training scheme for NTGPNs, developed and delivered by the South Yorkshire Primary Care Workforce & Training Hub (SY PCWTH) as the first part of the national GPN Fellowship scheme [32].

### Study aims and objectives

To examine the perspectives of the VTS trainees on a career in general practice regarding:

1. The culture of general practice in the UK.
2. ‘Readiness to practise’ as GPNs.
3. The development of a GPN career pathway.

## Study design

The study used a longitudinal, qualitative design, following the educational trajectory of a cohort of trainees’ over the 12-months of the programme. It used a constructivist approach, to enable the team to study the trainees’ perceptions of a career in general practice at key points throughout the programme.

### Research governance

Ethical approval was obtained from the Sheffield Hallam University (SHU) Research Ethics Committee (Ref: ER27858429), and SHU research governance protocols were adhered to throughout the study. All data were anonymised by the removal of any identifiable information, to maintain confidentiality and to ensure that no individual could be recognised in any subsequent report or publication.

All of the electronic data was held on a password-protected, encrypted network storage system that adheres to Home Office Standards of Data Security. These data will be kept for a minimum of seven years in accordance with SHU guidelines.

### Ethical issues and consent

Given that this was a study involving trainees as participants, great care was taken to avoid any perception of coercion. Particular emphasis was given to reassure the participants that (a) they had the right to refuse to take part and (b) they would not be disadvantaged if they chose not to take part.

As Sim & Waterfield [35] note, there are a number of ethical issues specifically related to focus groups. For example, the unpredictable nature of focus group discourse may give rise to problems with confidentiality, and also limit the extent to which potential problems can be identified during the consent process.

The dynamics within the group may also lead to some individuals dominating the discussion and thereby denying or denigrating other participants’ views. In addition, managing participants’ distress within focus groups is a challenge that needs to be considered.

It is clear that some of these ethical challenges can be addressed through a robust consent process, however efforts may need to be made to reinforce these issues closer to the actual focus group. This may be done in the form of a briefing immediately prior to the discussion, during the discussion itself, or in a debriefing immediately after the focus group has finished.

### Recruitment

The participants (n = 17) were recruited from the population of trainees (n = 21) undertaking the September 2020 VTS programme. A preliminary information session regarding the nature and purpose of the study was provided for the participants, with the opportunity to ask questions. Following the information session, interested trainees were invited to contact the nurse lead for the programme giving permission for their contact details to be passed to the study team. The resulting 17 participants were provided with an online information sheet and consent form to complete.

All of the participants identified themselves as female. Ten of the seventeen participants (59%) were new graduates. Of the seven participants (41%) who were not ‘new’ graduates, six came from secondary care and one from primary care. On average, the more experienced participants had been qualified for 4.8 years with a range of between 18 months and 7 years. Approximately half (53%) of the participants were aged between 20 and 29, with an age range of 23 years to 47 years.

### Data collection

The data were collected using online focus groups. The membership of each focus group was variable, dictated by the availability of the participants on the day. This meant that not all of the participants attended all of the focus groups, with an average of 12/17 participants in each focus group. With the agreement of the SYPCWTH team, data collection took place during time allocated for personal professional development. The initial question schedules focussed upon the participants’ experiences and were based upon a rapid review of the existing literature undertaken as part of the study.

In order to ensure participant safety during the COVID-19 pandemic, the focus groups were conducted online using Zoom© [36]. This software uses voice over internet protocol (VoIP)-mediated technology. The focus groups were facilitated by RL at 3-monthly intervals. With the participants’ consent the audio-visual content of the Zoom© sessions were digitally recorded and the audio transcribed. The video content was stored securely on the SHU ‘cloud’ and used for reference purposes by the team.

### Data analysis

In addition to capturing the video data from the VoIP technology, the audio data from the focus groups were downloaded from the ‘cloud’, and digitally transcribed. The data were cleaned and cross-checked for accuracy by RL and an early career researcher (ECR) colleague to make sure that the participants’ individual contributions were separated, anonymised, and categorised. The cleaned and cross-checked data were then analysed using recognised data analysis software (Quirkos©). Data analysis was carried out using Ritchie & Spencer’s ‘framework analysis’ [37]. This involves the systematic processing, sifting, charting, and sorting of material of all types. It also allows the integration of existing knowledge from previous research and policy into the analysis of the data [38].

## Findings

There were a number of key themes generated by the data (see Table 1). The timed series nature of the focus groups meant that the thematic analysis of the data was linked to the time at which the focus group took place. The questions were designed to explore the VTS trainees’ views on a GPN career pathway.

**Table 1:** Key themes

Theme:	Sub-theme(s):
Pathways into general practice: opening doors to a new career	• *‘The world before the VTS’* • *‘You always seem to need experience first’*
Learning to become a GPN: expectations and transitions	• *‘I want to be able to hit the ground running’* • *‘The need for ongoing support’*
The future GPN: the need for a ‘proper’ career pathway	• *‘The GPs don’t see it as a priority’* • *‘The need for a fundamental rethink’*

### Pathways into general practice: opening doors to a new career

The first focus group took place within a month of the start of the programme. The initial questions related to the participants’ experiences of ‘getting into’ general practice. They were asked about their experiences of primary care and the nature of any general practice content in their undergraduate courses.

#### The world before the VTS…

The paucity of specific general practice education in the UG curriculum has been well-documented [16]. There has long been an assumption that the majority of new graduate nurses would work in secondary care following graduation. This participant (T10) outlined what ‘the world before VTS’ was like for her. She discussed the lack of general practice content in her own undergraduate curriculum and the impact on her career. She had been qualified for over 10 years and noted:

*When I qualified you just went onto a ward… that’s what we all did back then. There was nothing in the course about it* [being a GPN] *so it never occurred to me at all… it was all hospital stuff and ward work…*.

T10 went on to say:

I wish I’d had all this when I qualified… it would have made such a difference to me and the decisions I made at the time.

Despite the impact of the ATPS scheme on increasing the number of student nurse placements in general practice, there was still a lack of attention paid to general practice in undergraduate nursing programmes. For example, participant (T8) noted that:

*“It’s almost as if it* [general practice] *didn’t exist on my course… We didn’t do much on it at all really at uni* [sic]*…”*.

#### You always seemed to need experience first…

In addition, there was still an antipathy to general practice as a suitable ‘first post’ job for a newly qualified nurse:

*“They said to me… you have to go into secondary care first… they don’t take* [sic] *newly qualifieds so go spend a year on a ward first… get some experience under your belt”* (T11).

Consequently, would-be GPNs were being put off from applying for GPN posts. In addition, some of the cohort had already applied for GPN posts prior to starting the programme:

*“They always want you to have experience… so how do you get it… it’s a vicious circle… that’s the problem… no experience no interview* [sighs]” (T3).

This participant (T9) explained the issues:

*Every* [GPN post] *job I applied for asked for experience… I didn’t get a single interview*.

One participant (T2) asked a very reasonable rhetorical question. She asked me:

*Why do you need experience when other areas like ICU don’t ask for it… it doesn’t make any sense to me?*

The answers to this question are complex, multi-layered, and may lie at the root of the current GPN recruitment and retention crisis.

### Learning to become a GPN: expectations and transitions

The second online focus group took place approximately three months after the start of the programme. The participants were asked to reflect back upon their expectations for the VTS programme when they started.

#### I want to be able to hit the ground running…

The programme was designed to ensure that the trainees were able to function as ‘fully formed’ GPNs by the end of this first year. This participant (T13) summed up her thoughts on the nature and purpose of the programme very succinctly:

I was clear that when I finished it… I wanted to be able to hit the ground running….

She also noted:

It seems to give you everything you need within a year which is great… it’s all there for me in one go….

There was some apprehension amongst the participants, particularly those from secondary care. This participant (T11) said:

*I was really quite nervous to begin with… coming from a ward, it* [general practice nursing] *was all new to me and I felt a bit out of my depth to begin with*.

The participants had clear expectations regarding course content. The more clinically focused sessions seemed (to the participants) to capture the nature and essence of the GPN role.

*“I was really looking forward to the various long-term conditions sessions… and they were great… I learned a lot of really important stuff there… I see that as the main bit* [sic] *of my role going forward”* (T16).

The participants appreciated being taught by expert clinicians, and clearly valued their knowledge, experience, and clinical credibility. Learning the technical skills required for general practice was also seen as an important aspect of the course. One of the more experienced participants (T12) from (adult) secondary care said:

*I was a bit nervous about some of the practical skills and worried that I would find things like the baby ‘vaccs and imms’* [sic] *quite difficult… I needn’t have worried though they made it seem easy somehow*.

#### The need for ongoing support…

The third focus group took place at the start of the second of the clinical placements, halfway through the programme. By this time, the participants were settling into life working as a GPN as well as being a trainee. Crucially, the trainees were all given ‘protected learning time’ for the duration of the programme.

*“It* [the protected time] *was a really positive thing for me… had I been employed full time in the usual way I think I would have struggled to cope with all the coursework…”* (T5).

When asked, the participants reported that they felt well-supported by the practice team(s) and were, in the main, provided with the opportunities to practice the skills that they had learned.

*“I ran my own clinics but felt that I could still ask questions if I needed to… she* [T10’s supervisor] *never made me feel silly for asking for help”* (T10).

This participant (T16) was extremely complimentary over both the support provided by her preceptor/mentor and the general practice team as a whole. She noted:

*“The whole team were really supportive…* C1 [my preceptor] *was a really good example of what I imagined a ‘good’ GPN to be… she was extremely knowledgeable and approachable… happy to share her knowledge too…”*.

However, the need for clinical supervision was an issue that clearly impacted upon the cohort of trainees as a whole. The more autonomous nature of the GPN role in comparison to ward work meant that the opportunity to ask clinically focused questions and to get feedback on their progress was seen as important by the participants:

*“I didn’t get much what you would call ‘proper’ clinical supervision… discussing cases an’ that* [sic]*… working on my own… it was hard going at times I could’ve done with a bit more really”* (T9).

There was agreement that clinical supervision needed to be ongoing, even after the VTS has finished. In particular, the new graduate participants raised fears over support in the year following the VTS. There was a justifiable concern that they would be simply ‘cast adrift’ in the second year of the Fellowship, which was then unfunded, and left to ‘get on with it’:

*“We all needed a lot of support in clinical practice during this first year… but it needs to continue afterwards… one year isn’t enough really… not for me anyway* (T16).

### The future GPN: the need for a ‘proper’ career pathway

As the participants came towards the end of the programme, their thoughts inevitably turned to the future and what would happen to them once the VTS finished. When asked for their thoughts on a long-term future in general practice, they were both articulate and thoughtful in their views. There was a lot of discussion regarding the need for, and the absence of, a clearly identified career pathway. The ad hoc nature of GPN education did not sit easily with the younger, new graduate participants. T14 noted:

*… If I’m going to stay in general practice, I need to be able to see clearly where I’m going… where I’m heading* [sic] *how I’m going to get there… and how I’ll know when I’ve got there*.

When asked about the future of the GPN role itself, there was an assumption from the younger participants that the future GPN would need to be more autonomous and highly skilled.

*“I think that GPNs will take on the more complex patients… those* [sic] *with multi-morbidities and their role will also become more supervisory in nature…”* (T17).

In this regard, the ANP (advanced nurse practitioner) role was seen as a desirable ‘end point’ by a number of the participants. They were slightly disparaging over what they called the ‘clinic nurse’ role and clearly wanted to progress quickly. One of the younger participants (T13) said with some emphasis:

*I don’t want to be just* [sic] *a clinic nurse… I watched the ANPs running their own clinics, seeing their own patients… prescribing medications… that’s what I want to do definitely*.

She did however go on to say:

How I’m going to get there is another question entirely… someone will have to pay for it… but who? I can’t….

The issue of funding for education and training was a recurrent theme throughout the study. The nature of general practice in the UK meant that GPN career progression was primarily a business decision for the GP partners.

#### The GPs don’t see it as a priority…

Although all of the participants had found employment as GPNs by the end of the programme, there seemed to be an inherent contradiction in the assumption that the GPs wouldn’t fund their continuing professional development (CPD), because they would leave as soon as they had obtained the new qualifications:

*“I don’t think the GPs see it* [CPD] *as a priority… so there will be no real support for me after the foundation year which is a real shame”* (T4).

The tension between general practice as small to medium enterprises (SMEs) and the cost of education was seen as a barrier to GPN career progression [12–18]. The participants were worried that in spite of the clear benefits of the VTS programme, the barriers to professional development would remain. Whilst the VTS was seen as an important first step, any further CPD would be difficult to access:

*“Often nurses get to a particular stage in their development and want more… whatever that may be… which might not fit in with the practice business plan… so they have to move to another practice to continue their development which seems really short-sighted to me…”* (T15).

She (T15) went on to say:

… So the surgery won’t increase their salary… then they leave… it kind of becomes a self-fulfilling prophesy.

#### The need for a fundamental rethink…

When asked to amplify their comments on the provision of CPD, a number of the participants highlighted the somewhat arcane culture that still existed within general practice. This new graduate participant was very clear that there were entrenched cultural barriers to her future career as a GPN:

*“I still don’t get any sense of a ‘proper’ career pathway* [for GPNs] *… I think that the culture across general practice as a whole is still a huge barrier to our career advancement… the GPs just want us to be clinic room nurses at the moment*” (T13).

In addition, the absence of any formal professional development ‘infrastructure’ for GPNs was also highlighted by the participants. What was available to GPNs was unfavourably compared to what was currently provided for (medical) GP trainees. Having worked alongside GP trainees and seen what was provided for their medical counterparts; some of the GPN trainees were understandably frustrated. As T17 noted:

*Why can’t we have a proper GPN training course like the one they have for the doctors… I’ve seen what they get… they seem to have it sorted don’t they… but then they always seem to look after their own?”*

This participant (T4) agreed. She said:

How are they going to keep young ones like me? It’s got to be down to the GPs and the practice managers to change the way they think… but it needs to happen soon… or I won’t be staying long that’s for sure.

In spite of all this, the VTS programme was seen as an important first step towards a professional development pathway for GPNs, albeit with some small caveats:

*“It* [the VTS] *has begun to change things that’s for sure… but we still need a fundamental rethink… the culture needs to change but that’s easier said than done… isn’t it?”* (T15).

## Discussion

Worldwide, a significant amount of income for individual practices is generated by GPN activity [24]. In the UK, as elsewhere, this is done through ‘fees-for-service’ payments. In the UK these are known as the Quality Outcomes Framework (QOF) payments. The framework provides targets for LTC surveillance and management [39]. Consequently, GPs have preferred to recruit already-experienced nurses when there is a vacancy, rather than invest the time and money in the education and training that new GPNs will inevitably need to take up this role [18].

There is still evidence therefore of a GPN recruitment ‘merry go round’ in the UK, in which GPNs are simply ‘poached’ from other GP practices as required. As a result, there has been little incentive for new graduate nurses to consider applying for a GPN post [19]. It may be argued that maintaining the status quo has also suited GPs on a hegemonic, patriarchal level. Since this cohort of GPNs has consisted almost exclusively of mature women who were further into their careers and looking for a more ‘family friendly’ work environment, these already-experienced nurses would be less likely to demand promotion and a commensurate increase in salary [40].

All the evidence internationally [13, 21, 22, 28–33] shows that a continued emphasis upon the recruitment of already-experienced practice nurses has significantly hindered the appointment of new graduate nurses to general practice and as a consequence, the establishment of a career pathway for GPNs. As a consequence of the shift in emphasis from secondary to primary care, the prospects for NTGPNs have slowly begun to improve. Worldwide the need to recruit and retain NTGPNs to address the shortage has driven primary care, as a whole, to explore options for recruiting more NTGPNs [29–31]. However, in spite of this, access to general practice for new graduate nurses still remains an issue [34].

The expansion of general practice placements for student nurses [17], together with a belated increase in focus upon primary care within the UG curriculum [19], have been key in addressing the issue of access to general practice. The ‘world before the VTS’ was characterised by the participants in terms of a lack of exposure to primary care nursing, as students. By increasing both primary care content in the UG curriculum and general practice placement capacity, HEIs provide both student nurses and GPs with the means to make informed judgements regarding the perceived suitability of general practice for NTGPNs [21]. Whilst the delivery of increased placement capacity has improved the numbers of NTGPNs [24], it has not provided the numbers of primary care staff required to address the shortfall. As a number of the participants noted, GPs remained reluctant to employ NTGPNs due to the perceived need for previous experience, as outlined above.

There were a number of issues that needed to be addressed. The financial and logistical difficulties inherent in providing cover for staff undertaking training adversely affected the likelihood of the GPNs being released to study [31, 32]. The need to address the issue of funding was crucial. By funding the programme, the GPs were reimbursed for the trainees’ time and their supervision on placement. It was clear that many previous attempts to provide GPN education had failed as a result of a lack of funding [32]. Previous attempts at developing a formal programme of GPN education in the UK, linked to a career pathway, had always foundered over various disagreements regarding the funding of that education, amidst the vagaries of the culture in which general practice operates [25, 26].

Evidence has shown that the ‘transition to primary care’ programmes provide a bridge between increasing access to general practice for UG students and increasing the number of GPNs in post. In Australia, the development of transition to primary care professional programmes have been reported to increase levels of confidence and competence in Australian NTGPNs, within their first year of general practice. Similarly, the VTS programme described here was also adjudged by the trainees, albeit anecdotally, to have provided them with the skills that they needed to ‘hit the ground running’ as a GPN [32]. The desire to ‘hit the ground running’ was seen as an important aspect of this particular programme.

One of the positive aspects of the VTS programme was the protected time afforded to the trainees. This provided the trainees with the opportunity to learn and then practise key skills in a timely manner, to manage their own diaries, to reflect, and to organise clinical and peer supervision. Although ad hoc support was provided for the trainees whilst on placement, working ‘solo’ in a largely autonomous setting such as general practice raised some (valid) concerns for the younger, newly graduated participants [41]. Adjusting to a more isolated working environment in general practice required significant, ongoing support. Inevitably, the new graduate trainees’ need for clinical supervision was greater than some of the more experienced trainees, and there was some concern that this was not always forthcoming [42]. It may be argued that the increased need for supervision amongst some of the younger trainees was not picked up by the supervisors, who were used to supervising NTGPNs who were already very experienced, albeit in many other clinical contexts [43].

Looking further ahead, the participants were also concerned that once the funding for the VTS finished, so would their educational opportunities and the supervisory support that went with it [42]. Over the years there have been various attempts to produce ‘competency frameworks’ for GPNs [33, 34, 44–46]. Worldwide, the use of competency frameworks to ‘map’ GPN activity has been useful in articulating the GPN skillset and setting standards. For example, supported by the Australian government, the Australian Primary Care Nurses Association (APNA) developed a framework for advancing general practice nursing [33] from UG student to Nurse Practitioner (NP). This framework, supported by the Australian government, is an acknowledgement of the need for a career pathway. Similarly, this need was highlighted by a number of the participants, as they pondered the development of their future career [25, 32, 44, 45]. Although the majority of participants were under the age of 30, having an identifiable career trajectory was a key concern for all of the trainees. There was a universal agreement that the culture of general practice needed to substantially change in order to facilitate this [45, 46].

The trainees were also clear that the prevailing attitude(s) towards the provision of continuous professional development for GPNs also needed to change. Unfavourable comparisons were made by a number of the trainees between what they saw as the comprehensive, well-resourced, and properly funded career pathway provided for GP trainees and that currently on offer for GPNs [45].

The independent ‘small business’ culture of ‘fees for service’ general practice makes the development of any GPN career pathway challenging. In the UK, the QNI paper on standards of education and practice [47] and the HEE/Skills for Health document ‘Primary Care and General Practice Nursing Career and Core Capabilities Framework’ [48] have similarly gone some way towards describing a putative GPN career pathway, however there is still a need to sustainably ‘operationalise’ any pathway financially at the local, regional, and national level. The NHSE GPN Fellowship programme [45], under which umbrella the SY VTS programme operates, was seen by the trainees as an important first step towards developing a nationally funded, sustainable, GPN education and career pathway.

### Limitations of the study

The study took place during the COVID-19 pandemic, which clearly had a significant impact upon the trainees’ experiences of the VTS programme. The enforced move from classroom learning to online learning and the use of VoIP technology will have affected the participants’ views of the programme per se, and the author has tried to take this into account. The small sample size, the focus upon one cohort from a single programme are all acknowledged as study limitations. In addition, the pragmatic nature of the study meant that it was not possible to use a ‘neutral’ facilitator. Although the facilitator (RL) was not part of the programme delivery team, it is acknowledged that this may be a potential source of bias.

## Conclusion

Changing the workforce culture within general practice nursing was/is never going to be easy. Despite the success of the various access schemes in changing attitudes within undergraduate nursing clinical placements, the number of newly qualified nurses accessing general practice as their first post destination has remained stubbornly low. The reasons for this are multifaceted. The lack of primary care content in UG curricula remains an issue, as does the need for GPNs to need previous experience.

The need to create a sustainable workforce ‘pipeline’ for general practice, however, has never been more critical. If this is to be successful, new, younger, NTGPNs must be able to see general practice as both a suitable ‘first post’ destination and a viable career option in the longer term. Therefore, there must be clearly defined career pathways with the necessary, associated educational infrastructure to support GPNs in their professional and career development. As a successful first step towards the development of a sustainable post-qualification GPN career pathway, transition to general practice programmes such as this must be fully embedded into the infrastructure and culture of general practice, and the necessary funding to ensure their long-term future must be guaranteed.

## Data Availability

The anonymised datasets used and analysed for the current study are available from the corresponding author (RL) upon reasonable request.

## Abbreviations

GPN: General Practice Nurse
UK: United Kingdom
LTC: Long term condition
QNI: Queen’s Nursing Institute
COVID-19: Coronavirus disease 2019
NHS: UK national health service
GP: General practitioner
HEE: Health Education England
CPEN: Community education provider
ATP: Advanced training practice
NTGPN: New to general practice nurse
SY: South Yorkshire
VTS: Vocational training scheme
SHU: Sheffield Hallam University
VoIP: Voice over internet protocol
CPD: Continuous professional development
SME: Small to medium enterprises
QOF: Quality outcomes framework
NHSE: NHS England
